# A problem of proportions: estimates of metabolic associated fatty liver disease and liver fibrosis in Australian adults in the nationwide 2012 AusDiab Study

**DOI:** 10.1038/s41598-022-05168-0

**Published:** 2022-02-04

**Authors:** Ann M. Farrell, Dianna J. Magliano, Jonathan E. Shaw, Alexander J. Thompson, Catherine Croagh, Marno C. Ryan, Jessica Howell

**Affiliations:** 1 Department of Gastroenterology, St Vincent’s Hospital Melbourne, 41 Victoria Pde, Melbourne, 3065 Australia; 2 University of Melbourne, Melbourne, Australia; 3 Baker Heart and Diabetes Institute, Melbourne, Australia; 4 Disease Elimination, Burnett Institute, Melbourne, Australia; 5 Department of epidemiology and preventive medicine, Monash university, Clayton, 3168 Australia

**Keywords:** Non-alcoholic fatty liver disease, Epidemiology, Public health

## Abstract

Metabolic Associated Fatty Liver Disease (MAFLD) is the most common cause of liver disease in Australia, but prevalence data are limited. We aimed to describe the frequency of alanine aminotransferase (ALT) elevation, and MAFLD within a large prospective Australian cohort. Cross-sectional analysis of the 2012 survey of the Australian Diabetes, Obesity and Lifestyle (AusDiab) study which included 4747 Australian adults (aged 34–97 yrs) was performed. Frequency of ALT elevation (men ≥ 40 IU/L, women ≥ 30 IU/L) and MAFLD (Fatty Liver Index (FLI) > 60 alongside metabolic risk factors) was determined and risk of advanced fibrosis stratified using the BARD score. Elevated ALT was found in 13% of the cohort, including 22% of people with diabetes, 18% with obesity, and 17% with the metabolic syndrome. 37% of the cohort had MAFLD, and those with MAFLD were more likely to be older (OR 1.01 per 1 year (95% CI 1.00–1.02)), male (OR 1.37 (95% CI 1.17–1.59)), have ALT elevation (OR 3.21 (95% CI 2.59–3.99)), diabetes (OR 3.39 (95% CI 2.61–4.39)), lower HDL-C (OR 0.15 per 1 mmol/L (95% CI 0.12–0.19)), higher diastolic blood pressure (OR 1.05 per 10 mmHg (95% CI 1.05–1.06)), a sedentary lifestyle (OR 1.99 (95% CI 1.59–2.50)) and less likely to have tertiary education (OR 0.81 (95% CI 0.7–0.94) compared to those without MAFLD. Of those with MAFLD, 61% had a BARD score suggesting risk of advanced fibrosis and 22% had an elevated ALT. Over 10% of this Australian cohort had elevated ALT, and 37% had MAFLD, with many at risk for advanced fibrosis.

## Introduction

Metabolic associated fatty liver disease (MAFLD) is the most common cause of liver disease, with a quarter of the global population estimated to be affected^1^. Due to the high global prevalence and rapid rise in the incidence of MAFLD in low-middle and high income countries, MAFLD is emerging as a major threat to national and global health^1,2^. The term MAFLD encompasses a spectrum of liver diseases characterised by excessive hepatic fat accumulation in the absence of significant alcohol use or other secondary causes of steatosis^3^. Hepatic steatosis can lead to steatohepatitis (MASH), and progress to hepatic fibrosis and cirrhosis^1,2^. MAFLD has also become the most rapidly increasing cause of hepatocellular carcinoma (HCC)^4–6^. Despite this, to date there are no approved medical treatments for MAFLD, with weight loss the only intervention that has been proven to be of benefit^3,7^.

MAFLD is the most common cause of elevated aminotransferase levels in developed and developing countries, especially in primary care^7–10^. In particular, elevated serum alanine aminotransferase (ALT) levels account for a significant proportion of the indication for referrals to specialist liver services^11^. While elevated ALT indicates liver damage, it has also been shown in cross-sectional studies to correlate with obesity and features of the metabolic syndrome^12,13^. Additionally, prospective cohort studies have consistently demonstrated that elevated ALT levels are associated with increased risk of developing the metabolic syndrome and type 2 diabetes (T2DM)^14–17^.

To date, there are limited data regarding the prevalence of elevated ALT and MAFLD within the Australian population. Previous Australian cohort studies have estimated the burden of MAFLD based on the frequency of ALT elevation, which occurred in 9–11% of adults and was associated with obesity, younger age, and features of higher cardiometabolic risk^12,13^. The recent Crossroads study performed in a single community in regional Victoria found MAFLD prevalence to be 36% using the fatty liver index, affecting a higher proportion of men and those with metabolic risk factors^18^.

In this cross sectional analysis, we describe the frequency of ALT elevation and MAFLD, using non-invasive diagnostic markers of hepatic steatosis in a large population-based Australian cohort. Additionally, we aimed to identify factors independently associated with an elevated ALT and to describe the proportion of MAFLD patients at risk of having advanced fibrosis.

## Methodology

### Study design

We undertook a cross-sectional analysis of individuals who had liver function tests (LFTs) performed in the 2012 survey (3rd follow up) of the Australian Diabetes, Obesity and Lifestyle (AusDiab) study. AusDiab is a nationwide population-based study, designed to investigate the prevalence of diabetes, obesity, hypertension and kidney disease in Australian adults(age ≥ 25 years). The original cohort were selected to be representative of the general Australian population at the time. The AusDiab study design, and methodology has been detailed elsewhere, with the baseline data collection performed in 2000, and follow up surveys in 2005 and 2012^19,20^. The sampling selection of the 2012 cohort is outlined in Supplementary Figure 1. This research was approved by the Human Research Ethics Committee of St Vincent’s Hospital Melbourne and all participants of the AusDiab study provided informed consent. All methods were conducted in accordance with the relevant guidelines and regulations.

LFTs were performed for the first time in this cohort in 2012, and other variables obtained in the 2012 survey included measures of obesity (body mass index (BMI) and waist circumference (WC)), and markers of metabolic health including blood pressure, lipid profiles, oral glucose tolerance tests and haemoglobin A1c (HbA1c).

### Data collection

Blood sampling was performed following an overnight fast (≥ 8 h) and blood specimens were collected in fluoride/oxalate tubes for glucose, and serum separator tubes for LFTs and lipids, which were centrifuged with serum/plasma separated on-site. The samples were transported to the central laboratory that day, however when not possible, were immediately frozen for transport. All analyses were conducted at a central laboratory (Healthscope Pathology, Victoria).

Diabetes was classified as a fasting plasma glucose of ≥ 7.0 mmol/l or two-hour post-load plasma glucose level of ≥ 11.1 mmol/l or receiving treatment with an antidiabetic agent as per the World Health Organization (WHO) guidelines^21^. Metabolic syndrome was defined using the International Diabetes Federation (IDF) guidelines^22^. Overweight and obesity were defined using the WHO classification for BMI (overweight 25–29 kg/m^2^, obese > 30 kg/m^2^) and WC (overweight F > 80 cm/M > 94 men, obese F > 88/M > 102 cm), and physical activity and sedentary behaviour were assessed by questionnaire^23^. Alcohol intake was obtained from self-administered Food Frequency Questionairres (FFQ) which was developed and validated by the cancer council of Victoria^24^. Excess alcohol intake was defined as ≥ 30 g per day for men and ≥ 20 g per day for women as per the EASL guidelines^3^.The definition of elevated ALT is not consistently defined in the literature, and can vary significantly between studies. In the largest Australian cohort to date (the Australian Health Survey (AHS) study), ALT elevation was defined as ≥ 40 IU/L in men and ≥ 30 IU/L in women^13^. We defined ALT elevation by this same threshold in order to present data comparable local data.

### Definition of MAFLD and risk of advanced fibrosis

MAFLD was defined using the diagnostic criteria proposed by Eslam et al., with hepatic steatosis determined using the Fatty Liver Index (FLI) and MAFLD diagnosed in those with steatosis alongside at least one of the following: BMI ≥ 25, presence of T2DM or BMI < 25 with at least two metabolic risk factors^25^. Using the Fatty Liver Index (FLI), a diagnostic algorithm incorporating BMI, WC, serum triglyceride and gamma-glutamyl transferase (GGT), score of > 60 had been determined as the threshold for detecting hepatic steatosis in Caucasian cohorts (AUROC 0.84)^26–29^.Within the criteria for metabolic risk factors, data were not available for two of the seven criteria: high-sensitivity C-reactive protein (hs-CRP) and the homeostasis model assessment of insulin resistance score (HOMA-IR). The BARD score (BMI ≥ 28 = 1 point, AST/ALT ratio (AAR) of ≥ 0.8 = 2 points, type 2 diabetes mellitus (T2DM) = 1 point) was used to identify those with MAFLD at low risk of advanced fibrosis. A score of ≥ 2 is associated with advanced fibrosis, while a score of < 2 has a high negative predictive value for ruling out significant fibrosis of 96%^30^.

### Statistical analysis

Baseline (2012) characteristics were compared between those with and without an elevated ALT. The distribution of data was determined using the Shapiro-Francia test, with non-parametric data presented as median with interquartile range, proportions compared using the chi-squared test, and comparison of continuous variables performed using Wilcoxon rank sum test for those with a non-parametric distribution. For variables with highly skewed distribution (serum triglycerides), log transformation was performed. Odds ratios were calculated for the association between elevated ALT levels and age, gender, smoking status, BMI, WC, diabetes, systolic and diastolic blood pressure, triglycerides, low-density lipoprotein cholesterol (LDL-C), high-density lipoprotein cholesterol (HDL-C), metabolic syndrome and exercise levels. An explanatory multivariable logistic regression model was developed using backward elimination and likelihood ratio testing, including variables that were associated with elevated ALT levels (*p* value < 0.1) on univariable analysis. Gender was not included in the multivariable ALT models due to implied bias of the gender specific ALT thresholds used. BMI and diagnosis of the metabolic syndrome were not included due to collinearity with other included variables (BMI collinear with WC and metabolic syndrome with the individual components of this diagnosis e.g. blood pressure). Where a linear test for trend was confirmed, ordinal variables were included as continuous variables in the model. The same modelling steps were performed for the association between MAFLD and age, gender, smoking, diabetes, systolic and diastolic blood pressure, LDL-C, HDL-C, ALT elevation at the higher threshold and exercise levels. Analysis was performed using Stata 15 software (StataCorp. 2017. Stata Statistical Software: Release 15. College Station, TX: StataCorp LLC).

### Ethics

This study has ethical approval through St Vincent’s Hospital Melbourne (LRR 225/20, project ID 69047).

## Results

### Baseline cohort and characteristics

The AusDiab study recruited 11,247 adults aged 25 and older for the baseline survey performed in 1999/2000, with 4747 returning for the 3^rd^ follow up in 2012. Among these 4747 participants, the median age was 60 years (IQR 53–68), 55% were female, 10% had T2DM, and two-thirds were overweight (41%) or obese (27%) according to BMI. The majority of participants identified as Caucasian ethnicity (93%), were born in Australasia or the United Kingdom including Northern Ireland (90%), and 66% lived in metropolitan capital cities.

### Elevated ALT is associated with many features of the metabolic syndrome

Elevated ALT (≥ 40 IU/L in men and ≥ 30 IU/L in women) was found in 13% of the cohort (n = 597), including 22% of people with diabetes, 20% of obese participants, and 17% of those with the metabolic syndrome. Distribution of clinical variables between those with and without elevated ALT levels is shown in Table 1. Those with elevated ALT were younger (57 years vs 61 years, *p* ≤ 0.001), with a higher median BMI (29 vs 27, *p* ≤ 0.001), more T2DM (17% vs 9%, *p* ≤ 0.001), and a greater proportion met the criteria for the metabolic syndrome (53% vs 38%, *p* ≤ 0.001) compared to those without ALT elevation.

**Table 1:** Metabolic and biochemical profiles of participants according to ALT level: the AusDiab study.

	Normal ALT	Elevated ALT ^†^	Crude OR (95% CI)	Adj OR (95% CI) ^‡^
Age (years/OR per 1 year)	61 (53–69)	57 (51–63)	0.97 (0.96–0.97)	0.96 (0.95–0.96)
**Gender**				
Female	55% (n = 2290)	57% (n = 338)	1 (reference)	
Male	45% (n = 1860)	43% (n = 259)	0.94 (0.79–1.12)	0.64 (0.53–0.78)
Smoker (vs non-smoker)	6% (n = 230)	5% (n = 26)	0.77 (0.51–1.17)	
BMI (kg/m^2^/OR per 1 kg/m^2^)	27 (24–30)	29 (26–33)	1.08 (1.06–1.09)	^§^
Waist circ (cm/OR per 1 cm)	93.5 (84–103)	99.3 (90–111)	1.03 (1.02–1.04)	1.02 (1.02–1.03)
T2 Diabetes (vs no T2DM)	9% (n = 360)	17% (n = 104)	2.22 (1.75–2.82)	1.89 (1.43–2.49)
Systolic BP (mmHg/OR per 10 mmHg)	128 (116–142)	130 (118–142)	1.00 (1.00–1.01)	
Diastolic BP (mmHg/OR per 10 mmHg)	72 (65–79)	76 (69–83)	1.03 (1.02–1.04)	1.01 (1.01–1.02)
Triglycerides^¶^ (mmol/L/OR per 1 mmol/L)	1.1 (0.8–1.6)	1.4 (1–1.9)	2.23 (1.89–2.64)	1.69 (1.39–2.06)
LDL (mmol/L/OR per 1 mmol/L)	3 (2.4–3.6)	3 (2.4–3.7)	1.05 (0.95–1.16)	
HDL (mmol/L/OR per 1 mmol/L)	1.5 (1.2–1.8)	1.4 (1.1–1.7)	0.51 (0.41–0.63)	
Metabolic syndrome (vs no metabolic syndrome)	38% (n = 1436)	53% (n = 297)	2.03 (1.7–2.45)	^§^
**Exercise**				
Sufficient	64% (n = 2585)	58% (n = 336)	1 (reference)	1 (reference)
Insufficient	25% (n = 1019)	26% (n = 152)	1.15 (0.94–1.41)	0.97 (0.78–1.21)
Sedentary	11% (n = 440)	16% (n = 92)	1.61 (1.25–1.07)	1.32 (1.01–1.73)

^**†**^Elevated ALT defined at > 40 IU/L in men and > 30 IU/L in women.

^**‡**^Multivariable analysis performed by logistic regression examining ALT elevation adjusted for age, gender, WC, T2DM,diastolic blood pressure, triglycerides and exercise.

^§^Not included in multivariable model due to collinearity: BMI collinear with WC, metabolic syndrome collinear with multiple included variables (WC, lipids, blood pressure).

^¶^Log transformed variable used for crude and adjusted OR.

**Figure 1:**
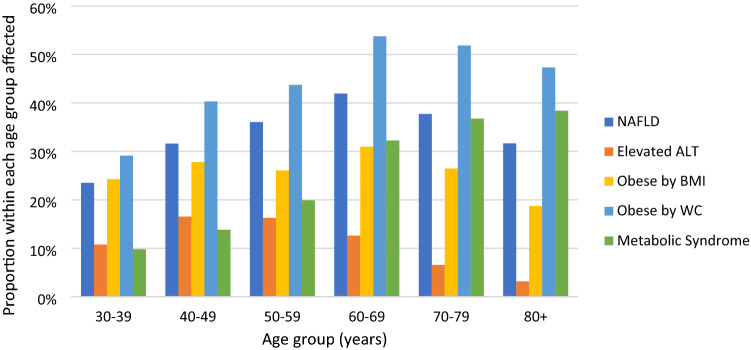
Frequency of mafld, alt elevation, obesity and the metabolic syndrome by age groups.

On multivariable logistic regression, female gender (OR 1.56, 95% CI 1.27–1.9), higher diastolic blood pressure (OR 1.01 per 10 mmHg, 95% CI 1.01–1.02), elevated triglyceride levels (OR 1.69 per 1 mmol/L, 95% CI 1.39–2.06), T2DM (OR 1.89, 95% CI 1.43–2.49), sedentary lifestyle (OR 1.32, 95% CI 1.01–1.73) and greater WC (OR 1.02 per cm, 95% CI 1.02–1.03), were independently associated with an elevated ALT. The relationship between age and ALT was non-linear, with ALT elevation occurring most commonly in middle age (Fig. 1).

### MAFLD and markers of advanced fibrosis

37% of the cohort had MAFLD. Those with MAFLD were older (OR 1.01 per year, 95% CI 1.01–1.02), more likely to be male (OR 1.37, 95% CI 1.17–1.59), have T2DM (OR 3.39, 95% CI 2.61–4.39), lower HDL-C (OR 0.15 per 1 mmol/L, 95% CI 0.12–0.19) and a higher diastolic blood pressure (OR 1.06 per 10 mmHg, 95% CI 1.05–1.06) compared to those without MAFLD. A sedentary lifestyle (OR 1.99, 95% CI 1.59–2.50) and not completing any post-secondary education (OR 1.24, 95% CI 1.06–1.44) were also independently associated with MAFLD on multivariable logistic regression analysis (Table 2). There was no difference in the frequency of MAFLD between those living in metropolitan and regional or rural areas (36% vs 38%, p = 0.171).

**Table 2:** Crude and adjusted odds ratios of variables associated with mafld.

	MAFLD	No MAFLD	Crude OR (95% CI)	Adj OR (95% CI)^†^
Age (years/OR per 1 year)	61 (54–68)	60 (52–68)	1.01 (1.00–1.01)	1.01 (1.00–1.02)
Male gender (vs female)	58%	37%	2.32 (2.05–2.62)	1.37 (1.17–1.59)
Smoker (vs non-smoker)	6%	5%	1.20 (0.93–1.57)	
T2 Diabetes (vs no T2DM)	19%	4%	5.43 (4.37–6.76)	3.39 (2.61–4.39)
Systolic BP (mmHg/OR per 10 mmHg)	133 (122–146)	123 (113–137)	1.02 (1.02–1.03)	
Diastolic BP (mmHg/OR per 10 mmHg)	76 (70–83)	69 (63–76)	1.06 (1.06–1.07)	1.05 (1.05–1.06)
LDL (mmol/L/OR per 1 mmol/L)	3 (2.3–3.7)	3 (2.4–3.6)	1 (0.93–1.07)	
HDL (mmol/L/OR per 1 mmol/L)	1.3 (1.1–1.5)	1.6 (1.3–1.9)	0.10 (0.09–0.13)	0.15 (0.12–0.19)
ALT elevation	22%	7%	3.57 (2.98–4.28)	3.21 (2.59–3.99)
(≥ 40 IU/L and ≥ 30 IU/L vs no ALT elevation)				
**Exercise**				
Sufficient	55%	69%	1 (reference)	1 (reference)
Insufficient	29%	23%	1.57 (1.36–1.81)	1.43 (1.21–1.69)
Sedentary	16%	8%	2.34 (1.93–2.83)	1.99 (1.59–2.50)
Post-secondary education (vs primary/secondary education only)	32%	40%	0.72 (0.63–0.81)	0.81 (0.7–0.94)

^**†**^Multivariable analysis performed by logistic regression examining MAFLD by FLI adjusted for age, gender, diastolic BP, T2DM, HDL cholesterol, ALT elevation, exercise and level of education.

NB: BMI, waist circumference, triglycerides and GGT not included in the model due to collinearity as these variables are included in the FLI diagnostic algorithm.

Participants with ALT elevation (≥ 40 IU/L in men and ≥ 30 IU/L in women), were over 3 times more likely to have MAFLD (OR 3.57, 95% CI 2.98–4.28) compared to those without ALT elevation. Elevated ALT was found in 22% of those with MAFLD. The diagnostic criteria for MAFLD correlated strongly with the presence of hepatic steatosis by FLI, with only two participants with hepatic steatosis not meeting criteria for MAFLD due to BMI < 25 and the presence of only one metabolic risk factor. Excess alcohol consumption (≥ 30 g/day men, ≥ 20 g/day women) was found in 22% of those with MAFLD and 20% of the total cohort.

In those with MAFLD, the BARD score ruled out advanced fibrosis in 38%. 61% had a BARD score ≥ 2 identifying them as at possible risk of advanced fibrosis and a high BARD score was also found in 35% of those with MAFLD who also had an elevated ALT.

## Discussion

In this large Australian sample we found a high frequency of ALT elevation (13%) and MAFLD (37%); with ALT elevation more common than has previously been described among Australian adults in the community. The Australian Health Survey (AHS), and Busselton cohorts found the frequency of ALT elevation to be lower at 11% and 9% respectively, with the AHS study noting the same independent relationship with younger age, diabetes, higher triglycerides and greater WC, consistent with the existing international literature^12,13^. All three cohorts examined ALT in a cross-sectional manner at a single timepoint, however these previous cohorts were younger (mean ages being; 43–46 years in AHS, 52yrs in Busselton, 60yrs in AusDiab), with fewer participants with diabetes (4.3–6.9% in AHS vs 10% in AusDiab), or obesity (17% in Busselton vs 27% in AusDiab), all factors likely to affect the frequency of ALT elevation in each cohort. Additionally, the Busselton study used a higher ALT threshold, > 40 IU/L for both men and women, which may also contribute to these differences.

The proportion of adults with elevated ALT was shown to decrease with advancing age independent of gender, alcohol use and the metabolic syndrome in a large longitudinal study, which was thought to be the result of diminishing liver and skeletal muscle mass associated with aging^31^. Similarly, in our sample the frequency of ALT elevation decreased in those over the age of 60, despite features of the metabolic syndrome increasing, and MAFLD remaining constant in this age stratum. This age disparity shows that while ALT elevation is associated with similar metabolic risk factors such as diabetes, hypertension and dyslipidaemia, it is an imperfect surrogate marker for MAFLD as it may under recognise MAFLD in older patients and additional diagnostic methods are often required^17,32,33^.

Estimating the prevalence of MAFLD is difficult, partly due to the absence of a widely available, non-invasive and accurate diagnostic test. MAFLD has a reported global prevalence of 25%, with estimates based on composite data from a range of studies with heterogenous diagnostic criteria that include radiological methods such as ultrasound, ALT elevation or clinical scoring systems such as the FLI^1^. To date, there are limited data about the prevalence of MAFLD in Australia. We present the first MAFLD estimates using the criteria proposed by Eslam et al. alongside a validated diagnostic biomarker to detect hepatic steatosis in an Australia-wide cohort^28^. Our results are consistent with those of the Crossroads study, performed in a single area in regional Victoria, which found the age- and sex- standardized prevalence of MAFLD to be 36% using FLI^18^. Our cohort predominantly lived in metropolitan capital cities (66%), with no difference found in the rates of MAFLD compared to those living in regional and rural Australia, further informing our understanding of the frequency of MAFLD throughout the Australian population. Like the Crossroads study, our cohort was predominantly Caucasian, and the European threshold of FLI > 60 was used in both studies. FLI uses a combination of clinical and biochemical factors to develop a score with good accuracy for predicting the presence of radiologically-evident hepatic steatosis with an AUC-ROC of 0.85^26^. While FLI does not predict the degree of liver fat, and has predominantly been validated against ultrasound and MRI, it is a useful diagnostic tool in epidemiologic research, having been widely used in this setting. The MAFLD nomenclature has arisen due to the current variability in the diagnosis of fatty liver in clinical practice and the desire to recognise it as a spectrum of disease which can co-exist alongside other liver diseases such as alcohol. When applying the MAFLD diagnostic criteria to our cohort, data was not available for two of the seven criteria for metabolic risk in those with BMI < 25 (hs-CRP and HOMA-IR), however these are not widely used screening tests in clinical practice and are unlikely to alter our findings. These data offer useful clinical information about the degree of MAFLD in a large Australian cohort, enriched with older participants, in the absence of radiological population-based screening studies in Australia^34^.

The high proportion of MAFLD in our study, is likely to be reflective of the advanced age and high frequency of overweight and obesity (69%) and diabetes (10%) in our cohort. While this is higher than that found in the general Australian population aged 18 years and over (63% and 4% respectively), it was similar to those of similar age strata in the nationally representative Australian National Health Survey which was also performed in 2012^35^. The high frequency of ALT elevation and MAFLD in our cohort is concerning from a public health perspective. How we best identify individuals at greatest risk of advanced fibrosis, and therefore at the greatest risk of morbidity and mortality, is a priority. Non-invasive fibrosis estimation algorithms, are widely used to identify patients at risk of advanced fibrosis who should be referred for specialist hepatology review. We used the BARD score which was able to be performed with the information available, and has been validated in multiple cohorts to perform similarly to the more widely known MAFLD fibrosis score^36,37^. These non-invasive markers tend to be sensitive but less specific for advanced fibrosis, and MAFLD alongside elevation of ALT is a more commonly used clinical trigger for specialist referral, as ALT elevation in MAFLD is associated with a higher risk of developing cirrhosis^11,38^. A significant proportion of our cohort were found to have MAFLD with an elevated ALT (8%), although the BARD score ruled out advanced fibrosis in 38% of those with MAFLD. Those with an elevated BARD score would benefit from further investigation with another non-invasive marker of fibrosis or a second-line investigation such as elastography. Additionally, as the obesity epidemic continues to grow, with rates of overweight and obesity in Australia increasing by 4% between 2012 and the most recent national health survey data in 2018, the associated MAFLD burden is also likely to increase even further^35^. Future studies examining the true prevalence of MAFLD and advanced fibrosis in the Australian population incorporating multiple non-invasive markers of fibrosis, alongside radiological or histological criteria to validate these findings are warranted.

There are some limitations to this study. This is a cross-sectional analysis of a follow up survey conducted as part of the larger prospective AusDiab study, and a predominantly Caucasian cohort. As a result, there may be selection and survival bias inherent within the cohort, and our findings may not be generalisable to the wider Australian population or other more ethnically diverse groups. Additionally, information about other known liver disease was not collected in 2012. However this is less relevant when the definition of MAFLD is applied, which allows for co-existing liver diseases. Furthermore, the community prevalence of viral hepatitis in Australia is relatively low (1–2%), with high risk populations such as migrants from endemic regions and people who inject drugs likely under-represented in this cohort^39^. A strength of this study was the ability to incorporate diagnostic and prognostic MAFLD algorithms, and to our knowledge this is the first large Australia-wide study to estimate MAFLD and risk of advanced fibrosis using validated clinical algorithms. This cohort is older than previously reported population-based cohorts examining ALT elevation, contributing to our current understanding of the association between metabolic health and liver enzymes in the Australian population across the lifespan. This information is important in informing the public health response to the growing healthcare burden associated with MAFLD.

## Conclusion

The frequency of MAFLD and elevated ALT was high in this large nationwide Australian cohort, with these figures expected to rise with the growing obesity epidemic. These numbers are important in healthcare resource planning, as the demand is likely to exceed the capacity of specialist liver services in the future.

## Supplementary Information

Supplementary Figure 1.
