# Supplementary material for: A problem of proportions: estimates of metabolic associated fatty liver disease and liver fibrosis in Australian adults in the nationwide 2012 AusDiab Study
Source: Sci Rep. 2022 Feb 4;12:1956. doi: 10.1038/s41598-022-05168-0 (PMC8817026; doi:10.1038/s41598-022-05168-0)
Supplement: Supplementary file 1 — Supplementary Figure 1. [file 41598_2022_5168_MOESM1_ESM.docx]

### Supplementary material

Supplementary figure 1: Sampling frame for AusDiab follow-up in 2012
